# Effects of MASP-1 of the Complement System on Activation of Coagulation Factors and Plasma Clot Formation

**DOI:** 10.1371/journal.pone.0035690

**Published:** 2012-04-20

**Authors:** Katharina Hess, Ramzi Ajjan, Fladia Phoenix, József Dobó, Péter Gál, Verena Schroeder

**Affiliations:** 1 Department of Internal Medicine, Cardiology, University Hospital Aachen, Aachen, Germany; 2 Division of Cardiovascular and Diabetes Research, The LIGHT Laboratories, Multidisciplinary Cardiovascular Research Center, University of Leeds, Leeds, United Kingdom; 3 Institute of Enzymology, Biological Research Center, Hungarian Academy of Sciences, Budapest, Hungary; 4 University Clinic of Hematology and Central Hematology Laboratory, Hemostasis Research Laboratory, University Hospital and University of Bern, Bern, Switzerland; Institut National de la Santé et de la Recherche Médicale, France

## Abstract

**Background:**

Numerous interactions between the coagulation and complement systems have been shown. Recently, links between coagulation and mannan-binding lectin-associated serine protease-1 (MASP-1) of the complement lectin pathway have been proposed. Our aim was to investigate MASP-1 activation of factor XIII (FXIII), fibrinogen, prothrombin, and thrombin-activatable fibrinolysis inhibitor (TAFI) in plasma-based systems, and to analyse effects of MASP-1 on plasma clot formation, structure and lysis.

**Methodology/Principal Findings:**

We used a FXIII incorporation assay and specific assays to measure the activation products prothrombin fragment F1+2, fibrinopeptide A (FPA), and activated TAFI (TAFIa). Clot formation and lysis were assessed by turbidimetric assay. Clot structure was studied by scanning electron microscopy. MASP-1 activated FXIII and, contrary to thrombin, induced FXIII activity faster in the Val34 than the Leu34 variant. MASP-1-dependent generation of F1+2, FPA and TAFIa showed a dose-dependent response in normal citrated plasma (NCP), albeit MASP-1 was much less efficient than FXa or thrombin. MASP-1 activation of prothrombin and TAFI cleavage were confirmed in purified systems. No FPA generation was observed in prothrombin-depleted plasma. MASP-1 induced clot formation in NCP, affected clot structure, and prolonged clot lysis.

**Conclusions/Significance:**

We show that MASP-1 interacts with plasma clot formation on different levels and influences fibrin structure. Although MASP-1-induced fibrin formation is thrombin-dependent, MASP-1 directly activates prothrombin, FXIII and TAFI. We suggest that MASP-1, in concerted action with other complement and coagulation proteins, may play a role in fibrin clot formation.

## Introduction

The coagulation and complement systems are activated following external injury to protect the host from blood loss and infections. The simultaneous activation of coagulation and inflammatory processes after injury is a phylogenetically ancient adaptive response that can be traced back to early eukaryotic evolution [1]. A number of recent studies show direct interactions between the two systems [2], [3], among them are links between coagulation factors and mannan (or mannose) -binding lectin (MBL) associated serine proteases (MASPs) of the complement lectin pathway.

The lectin pathway of the complement system is activated by binding of the target recognition molecules MBL or ficolins to carbohydrates or N-acetylated groups, respectively, on the surface of cells or microorganisms. MBL and ficolins circulate in complexes with MASPs which autoactivate upon binding of MBL/ficolins to their target structures. Three MASPs and two related proteins are present in human plasma, arising from two genes by alternative splicing: Mannose-binding lectin-associated serine protease-1 (MASP-1) and its alternatively-spliced variants MASP-3 and MAp44, and MASP-2 and its alternatively-spliced variant MAp19. MAp44 and MAp19 contain no serine protease domain and hence lack enzymatic activity [4], [5]. Average plasma concentrations of MASP-1 have been estimated at 6 µg/ml (range 1–12 µg/ml) [6] and 11 µg/ml [7].

Upon activation, MASP-2 cleaves both C4 and C2 and thus induces further complement activation by generating the lectin/classical pathway C3 convertase C4b2b. Furthermore, MASP-2 cleaves prothrombin to generate active thrombin [8]. In contrast, the physiological roles of MASP-1 and MASP-3 remain subject of research although potential substrates have been identified. MASP-3 does not activate either C4 or C2. MASP-1 cleaves C2 but not C4 and therefore is not capable of generating C3 convertase alone [9], as confirmed by a study in MASP-2 knockout mice [10]. Direct activation of C3 by MASP-1 occurs with very low catalytic efficiency and is not of physiological relevance [4], [5]. However, MASP-1 has been suggested to act synergistically with MASP-2 to produce C3 convertase via C2 cleavage [11], and MASP-1 may even activate MASP-2 [12], [13]. A new role for MASP-1 in the alternative complement pathway has been proposed, by directly activating complement factor D [14], [15]. MASP-1 may also exert proinflammatory effects through releasing bradykinin from high-molecular weight kininogen [16].

Experiments with synthetic peptides and structure-function analyses based on its crystal structure have revealed that MASP-1 has a much broader substrate specificity than MASP-2 and a thrombin-like activity with similar substrate specificity [9], [17], [18]. Indeed, MASP-1 is able to cleave the main substrates for thrombin, fibrinogen and factor XIII (FXIII), and activate endothelial protease-activated receptor 4, albeit with a lower catalytic efficiency compared with thrombin [4], [19], [20]. Cleavage of the FXIII A-subunit and the fibrinogen β-chain by MASP-1 occur at the same site as proteolysis by thrombin, whereas the cleavage site in the fibrinogen α-chain is different [19], indicating potential differences in the mechanisms of fibrin formation by thrombin and MASP-1. Activation of MASP-1 in complex with L-ficolin or MBL was also associated with generation of a fibrin clot [21]. First evidence for an *in vivo* role of MASP-1 in coagulation has been recently documented in a study demonstrating that mice lacking MBL or MASP-1 have a prolonged bleeding time following tail tip excision [22]. Furthermore, it has been recently demonstrated in a mouse model of FeCl_3_-induced intra-arterial thrombogenesis, that MASP-1 plays a key role in thrombus formation *in vivo* [23].

So far, studies on MASP-1 interactions with clotting factors mainly employed purified systems and cleavage/activation products of fibrinogen and FXIII were observed by Western Blotting and HPLC, but the effects of MASP-1 on clot formation and fibrinolysis in plasma or the characteristics of the clot were not directly assessed. Fibrin clot structure can determine predisposition to thrombotic events [24], [25], which is related, at least in part, to fibrinolysis efficiency as clots with denser structure and thinner fibers are generally more difficult to lyse [26]. The relationship between clot structure and lysis is in turn related to incorporation of different plasma proteins into the fibrin network [27]. A common genetic variant of FXIII, Val34Leu, influences FXIII activation and cross-linking efficiency leading to altered fibrin structure [28], [29]. Because of its high allele frequency and impact on thrombotic risk, the FXIII Val34Leu polymorphism is of major interest when studying fibrin formation and structure in the context of vascular diseases, but it has so far not been taken into account in studies investigating MASP-1-induced FXIII activation. Finally, given the thrombin-like activity of MASP-1, it is possible that MASP-1 also interacts with another thrombin substrate, thrombin-activatable fibrinolysis inhibitor (TAFI), which has not been studied before.

Therefore, the objective of this work was to investigate the effect of MASP-1 on activation of thrombin-dependent coagulation factors and fibrin clot formation in plasma environment. The specific aims were to 1) investigate MASP-1-induced plasma FXIII activation and analyse the effect of FXIII Val34Leu polymorphism, 2) investigate fibrinogen cleavage and activation of prothrombin and TAFI by MASP-1, and 3) analyse MASP-1-induced plasma fibrin clot formation, structure and lysis by turbidimetric assays and scanning electron microscopy.

## Methods

### Recombinant MASP-1

A recombinant mutant R504Q MASP-1 catalytic fragment, prepared as described earlier [9], [18], was used in this study. It has a molecular weight of 45.5 kDa and consists of the three C-terminal domains complement control protein 1 and 2 followed by the serine protease domain (CCP1-CCP2-SP). The mutant is resistant to autolysis and preserves its enzymatic activity during prolonged incubation (estimated half-life of several hours in plasma at 37°C), otherwise it has the same enzymatic properties as the wild-type enzyme. To simplify we use the term MASP-1 for the R504Q rMASP-1 catalytic fragment.

### FXIII activation

FXIII activation was assessed using an incorporation assay [28]. Briefly, a microtiter plate was coated with 100 µl/well of fibrinogen (Sigma-Aldrich, Switzerland) at 40 µg/ml (0.1 µM) in tris-buffered saline (TBS; 40 mM Tris, 140 mM NaCl, pH 7.4). After blocking with 200 µl of 1% bovine serum albumin (BSA), plasma diluted 1/10 as FXIII source or purified FXIII was added followed by the activation mix to a final reaction volume of 100 µl. The activation mix contained 5-biotinamidopentylamine (EZ-Link® Pentylamine-Biotin, Thermo Scientific, USA), dithiothreitol (Sigma-Aldrich), and CaCl_2_, at final concentrations of 0.3 mM, 5 mM, 10 mM, respectively, and human thrombin (Calbiochem, Merck Biosciences, Switzerland) or MASP-1 at various concentrations (as indicated in the results section). The reaction was incubated at 37°C and stopped at different time points by addition of 200 mM EDTA. The biotinpentylamine incorporated into fibrin by FXIIIa was detected by streptavidine-alkaline phosphatase (final concentration 1 µg/ml; Sigma-Aldrich), developed with p-nitrophenylphosphate as substrate, and read at 405 nm, with 620 nm as reference wavelength, on a microplate reader.

Besides normal citrated plasma (NCP; local plasma pool), we used FXIII-depleted plasma (FXIII-DP; Kordia, The Netherlands), and prothrombin-depleted plasma (PT-DP; American Diagnostica, USA) to show effects independent from thrombin generation. In some experiments, 1 µM C1 esterase inhibitor from human plasma (C1-Inh; Sigma-Aldrich) was used. Recombinant FXIII-A Val34 and FXIII-A Leu34 variants were obtained from Zedira (Germany).

### Fibrin cross-linking by FXIIIa

Fibrin cross-linking analysis by SDS-PAGE was performed to confirm that fibrin cross-linking occurs in presence of MASP-1. We incubated 100 µl citrated plasma (normal citrated plasma NCP or FXIII-depleted plasma FXIII-DP) with 50 µl CaCl_2_ (final concentration 5 mM) and 50 µl thrombin (final concentration 0.075 U/ml) or MASP-1 (final concentration 5 µg/ml) at 37°C for 45 min. To two FXIII-DP samples purified FXIII (final concentration 10 µg/ml) was added. After incubation, samples were spun down at 10'000 g for 10 min, the supernatants were removed and the clots washed twice in 1 ml TBS. After the last wash, 800 µl Laemmli sample buffer (Bio-Rad Laboratories, Hercules, USA) with β-mercaptoethanol was added and the samples were boiled at 95°C for 15 min to solubilize the clots. Purified fibrinogen (Calbiochem; final concentration 0.2 µg/ml) was used as control. Twenty microliters were loaded onto a 7.5% Tris-Glycine Mini-Protean® TGX™ gel (Bio-Rad). The gel was stained with Bio-Safe™ Coomassie (Bio-Rad).

### Fibrinogen cleavage and activation of prothrombin and TAFI

Cleavage of fibrinogen and activation of prothrombin were studied in NCP and PT-DP. Plasma samples (400 µl) were incubated at 37°C for 30 min with 100 µl of either TBS buffer only (non-activated, negative control), human thrombin (Calbiochem) at a final concentration of 0.1 U/ml (2.8 nM), human FXa (Hyphen BioMed, Neuville-sur-Oise, France) at a final concentration of 0.1 µg/ml (2.2 nM) with 6 mM CaCl_2_, or MASP-1 at final concentrations of 20 µg/ml (440 nM), 10 µg/ml (220 nM), 5 µg/ml (110 nM), 2.5 µg/ml (55 nM). Samples were then spun down at 10'000 g for 10 min, and the supernatant was snap-frozen in aliquots. For confirmation of prothrombin cleavage in a purified system, 100 µl of human prothrombin (Hyphen BioMed) at 100 µg/ml (1.4 µM) were incubated at 37°C with FXa at a final concentration of 0.1 µg/ml (2.2 nM)±2.5 mM CaCl_2_ or MASP-1 at final concentrations of 20 µg/ml (440 nM), 10 µg/ml (220 nM), 5 µg/ml (110 nM), 2.5 µg/ml (55 nM). At 5, 10, 15, and 30 min samples were snap-frozen. F1+2 was measured using the Enzygnost® F1+2 ELISA kit (Siemens Healthcare, Marburg, Germany). FPA was determined with the Zymutest FPA ELISA kit (Hyphen BioMed).

TAFI activation in plasma was analysed with the Actichrome® TAFI activity kit (American Diagnostica Inc, Stamford CT, USA). NCP and PT-DP, diluted 1/25, were incubated for 20 min with an activation mix containing either thrombin-thrombomodulin complex (final thrombin concentration 80–100 nM) or MASP-1 at final concentrations of 50 µg/ml (1100 nM), 10 µg/ml (220 nM), or 2 µg/ml (44 nM). Non-activated plasma was assayed in parallel. After stopping the activation reaction, activated TAFIa was detected with a highly specific TAFIa substrate. The concentration of TAFIa was calculated as the difference between activated and non-activated plasma samples.

TAFI cleavage by MASP-1 was verified using SDS-PAGE. To investigate the dose-dependent effect of MASP-1 on TAFI, 200 µg/ml (3.45 µM) of purified TAFI (Enzyme Research Laboratories, South Bend, USA) were incubated for 30 min at 37°C with MASP-1 at final concentrations of 100 µg/ml (2.2 µM), 200 µg/ml (4.4 µM), 400 µg/ml (8.8 µM), or 800 µg/ml (17.6 µM), diluted in TBS (40 mM Tris, 140 mM NaCl, pH 7.4). To investigate the time-dependent effect of MASP-1 on TAFI, 200 µg/ml (3.45 µM) of purified TAFI were incubated for 30 min, 60 min, and 120 min at 37°C without and with 400 µg/ml (8.8 µM) MASP-1. To confirm MASP-1-induced TAFI cleavage at physiological concentrations and compare it to thrombin/thrombomodulin-induced TAFI cleavage, the following experiment was performed: In a reaction volume of 50 µl, we incubated 15 µg/ml TAFI with either 15 µg/ml rMASP-1 or 1.7 U/ml (50 nM) thrombin and 3 µg/ml (58 nM) thrombomodulin (Abcam, Cambridge, UK) at 37°C for 1 h. This was similar to a protocol by Bajzar et al. [30]. After the incubation steps, reactions were stopped by adding Laemmli sample buffer (Bio-Rad Laboratories, Hercules, USA) containing β-mercaptoethanol, followed by boiling at 95°C for 10 min. Samples were run on Any kD™ Mini-Protean® TGX™ gels (Bio-Rad) and gels were stained with Bio-Safe™ Coomassie.

### Turbidimetric clot formation and lysis assay

Plasma clot formation and lysis was measured using a method adapted from Carter et al. [31]. Briefly, 25 µl of citrated plasma and 75 µl of TBS (50 mM Tris, 100 mM NaCl, pH 7.4) per well were added to a 96-well microtiter plate (Greiner bio-one, Germany). In the lysis assay, the 75 µl of TBS contained tPA (final concentration 83 ng/ml (1.2 nM)) (Technoclone, Austria). Upon addition of 50 µl of activation mix containing MASP-1 at various concentrations (see results section) or 0.03 U/ml (0.8 nM) (final concentration) human thrombin (Calbiochem) and 2.5 mM (final concentration) CaCl_2_ in TBS, the plate was placed into a microplate reader and optical density at 340 nm was recorded at 37°C every 30 sec for up to 2.5 hours. We analyzed the parameters lag time (time between addition of activation mix and exponential increase in absorbance), maximum absorbance (corrected for baseline absorbance), and lysis time (time between maximum absorbance and return to baseline absorbance). The turbidimetric assays were performed in NCP and PT-DP (to exclude thrombin action).

### Scanning electron microscopy of plasma clots

Forty-five microliters of normal citrated plasma diluted 1/2 with TBS were clotted upon the addition of 5 ul activation mix containing CaCl_2_ (final concentration 5 mM) and either thrombin (final concentration 0.88 U/ml (24 nM)) or different concentrations of MASP-1 (final concentrations 1 µg/ml (22 nM), 5 µg/ml (110 nM) or 10 µg/ml (220 nM)). As described earlier [27], clots were made in specially devised small perforated plastic vessels, incubated in a moist chamber for 2 hours followed by washing with sodium cacodylate buffer and subsequently fixed for 30 mins in 2% glutaraldehyde. Clots were recovered and further processed by a stepwise dehydration with acetone gradient and sputter coated with platinum palladium. Samples were viewed and photographed using a field emission gun, environmental scanning electron microscope (FEI, Quanta 200F, Hillsboro, USA). Ten images from different areas at three different magnifications (5000×, 10000×, 30000×) were taken per clot and visually examined by several investigators blinded to the clotting agent. Fiber diameters were measured with image analysis software package ImageJ 1.44p (Wayne Rasband, National Institutes of Health, USA, http://imagej.nih.gov/ij). Ten fibers per image, corresponding to 100 fibers per clot, were measured. Fiber diameter data were normally distributed and compared between the four groups of clots (thrombin, MASP-1 10 µg/ml, MASP-1 5 µg/ml, MASP-1 1 µg/ml) by Oneway ANOVA with post-hoc Bonferroni analysis (SPSS Statistics software, version 17.0.0).

### Summary of statistical methods

Experiments were performed several times, as described for the individual experiments, and results are shown as mean and standard deviation (SD) unless stated otherwise. In figures, data points represent mean values with error bars representing SD. SPSS Statistics software, version 17.0.0 (SPSS Inc.) was used for statistical analyses. Associations between MASP-1 concentration and parameters of the turbidimetric clot formation and lysis assay, i.e. lag time, maximum absorbance, and lysis time, were assessed using bivariate correlation analysis and expressed as Pearson correlation coefficient. Data on fibrin fiber diameters obtained from SEM of thrombin and MASP-1 clots were tested for normal distribution using the Kolmogorov-Smirnov test. Fiber diameters were compared between the four groups using the Oneway ANOVA and pairwise comparisons were analyzed using the post-hoc Bonferroni method.

## Results

### FXIII activation

**Figure 1:**
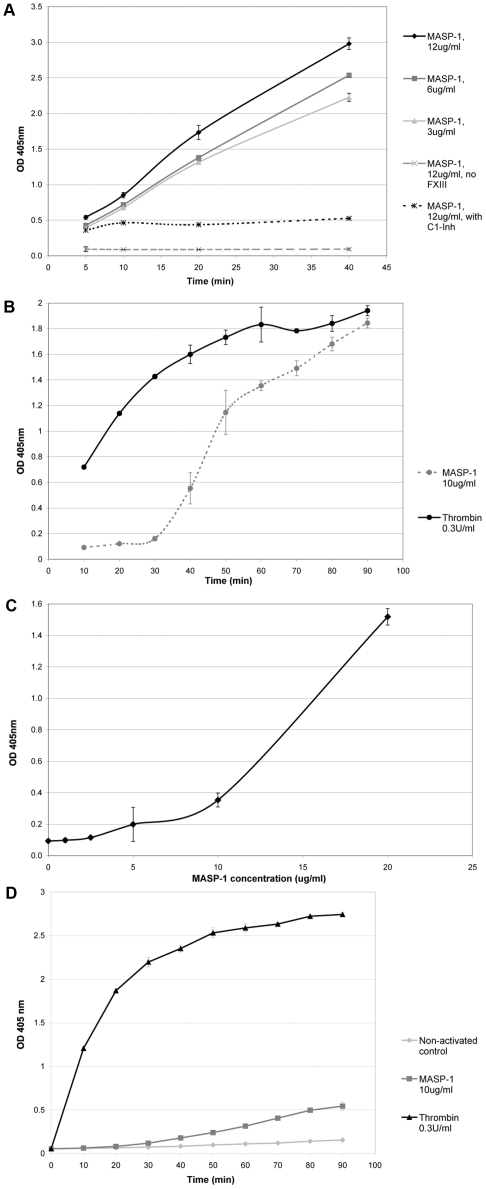
MASP-1-induced FXIII activation. Activation of FXIII was determined in a FXIII incorporation assay. (A) In a purified system, increasing MASP-1 concentrations were associated with increased FXIII activity, while C1-Inhibitor abolished MASP-1-induced FXIII activity. (B) In normal plasma, FXIII activation by MASP-1 occurred in a time-dependent (B) and dose-dependent (C) manner. (D) MASP-1 also activated FXIII in prothrombin-depleted plasma. Data points represent mean values from two (panels B and D) or three (panels A and C) experiments with error bars representing standard deviation.

In a purified system with 20 µg/ml (62 nM) of FXIII, MASP-1 induced FXIII-dependent biotin incorporation in a dose-dependent manner, whereas no incorporation was observed without FXIII or in the presence of 100 µg/ml (1 µM) of C1 inhibitor (Fig. 1a). In normal plasma (final dilution 1/25), MASP-1 induced FXIII-dependent biotin incorporation in a time- and dose-dependent manner (Fig. 1b, 1c). In the purified system, MASP-1-induced FXIII activation was evident at 5 min, whereas in plasma environment no effect was observed until 40 min, in contrast to thrombin which showed an early effect. No incorporation was observed in FXIII-depleted plasma (data not shown), whereas MASP-1 also induced FXIII-dependent biotin incorporation in prothrombin-depleted plasma (Fig. 1d), albeit less efficiently compared with normal plasma (Fig. 1b).

**Figure 2:**
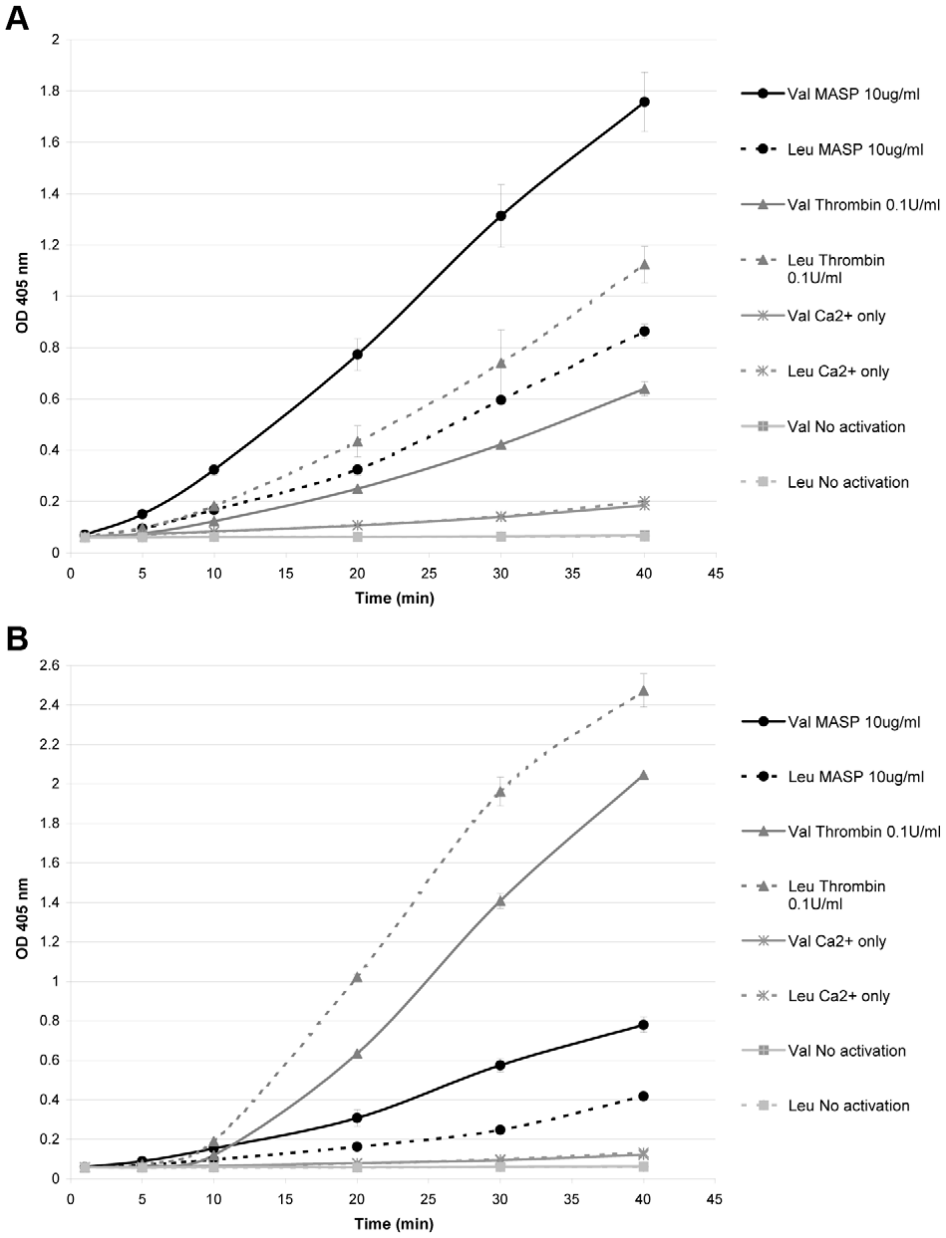
Activation of recombinant FXIII Val34 and Leu34 variants by MASP-1. (A) In a purified system, thrombin activated the Leu34 variant faster than the Val34 variant, whereas MASP-1 activated the Val34 variant faster than the Leu34 variant. (B) When added to FXIII-depleted plasma the same results in regard to Val34Leu variants were observed, but MASP-1-induced FXIII activation was less efficient compared to thrombin. Data points represent mean values from two experiments with error bars representing standard deviation.

When recombinant FXIII A-subunit Val34 and Leu34 variants were tested in TBS containing 1% BSA or in FXIII-depleted plasma, thrombin induced FXIII activity faster in the Leu34 variant compared to the Val34 variant, whereas the results were the opposite with MASP-1 which induced FXIII activity faster in the Val34 variant than the Leu34 variant (Fig. 2a, 2b). In the purified system, activation of FXIII Leu34 by thrombin or MASP-1 was similar, whereas FXIII Val34 activation showed large differences, with far superior activation achieved with MASP-1. In FXIII-DP, MASP-1 was again less efficient at activating FXIII, with both Val34 and Leu34 variant demonstrating higher activation by thrombin, in contrast to purified data. However, Val34 variant was still more efficiently activated by MASP-1 compared with Leu, in contrast to thrombin.

### Fibrin cross-linking by FXIIIa

**Figure 3:**
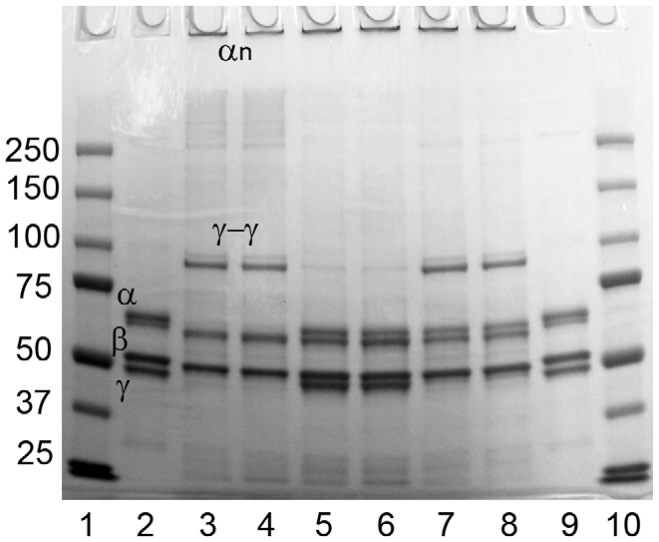
Fibrin crosslinking by FXIIIa. When thrombin or MASP-1 were incubated with NCP or FXIII-DP with added FXIII, γ-γ dimers and αn polymers were formed. No crosslinks were formed in FXIII-DP. Lane 1: Molecular weight marker. Lane 2: Purified fibrinogen. Lane 3: NCP+Ca^2+^+thrombin. Lane 4: NCP+Ca^2+^+MASP-1. Lane 5: FXIII-DP+Ca^2+^+thrombin. Lane 6: FXIII-DP+Ca^2+^+MASP-1. Lane 7: FXIII-DP+FXIII+Ca^2+^+thrombin. Lane 8: FXIII-DP+FXIII+Ca^2+^+MASP-1. Lane 9: Purified fibrinogen. Lane 10: Molecular weight marker.

Fig. 3 shows fibrin cross-linking by FXIIIa. Upon activation with thrombin and Ca^2+^ (lane 3), the fibrinogen Aα-chain (labelled α) was cleaved and partly formed high-molecular-weight αn polymers, the fibrinogen Bβ-chain (labelled β) was cleaved, and the fibrinogen γ-chain (labelled γ) completely disappeared to form γ-γ dimers. When NCP was incubated with MASP-1 (lane 4), the same cross-linking pattern appeared. No cross-linking was seen in FXIII-DP (lanes 5 and 6), but could be restored by addition of FXIII (lanes 7 and 8) confirming that this process was depending on FXIII.

### MASP-1 and activation/cleavage of prothrombin, fibrinogen, and TAFI

**Figure 4:**
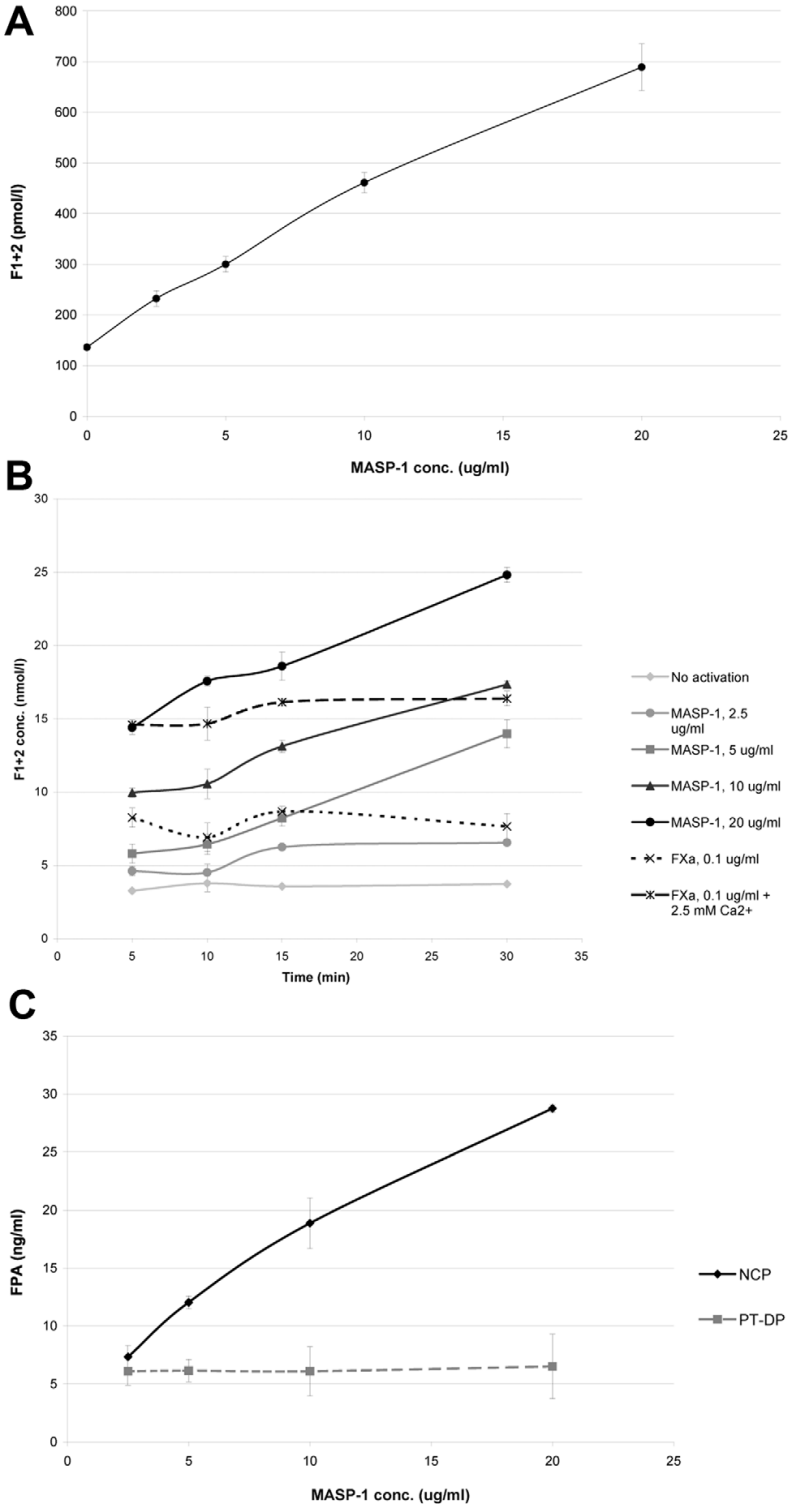
Activation of prothrombin, fibrinogen, and TAFI. (A) MASP-1 induced generation of the activation marker prothrombin fragment F1+2 in normal plasma. (B) Dose- and time-dependent prothrombin cleavage by MASP-1 was confirmed using purified proteins. (C) MASP-1 was associated with generation of the activation marker fibrinopeptide-A (FPA) in normal plasma (NCP) but not in prothrombin-depleted plasma (PT-DP). Data points represent mean values from two experiments with error bars representing standard deviation.

When MASP-1 was added to NCP, a dose-dependent generation of the prothrombin activation product, prothrombin fragment F1+2, was observed, although it was far less efficient than FXa (Table 1, Fig. 4a). We confirmed prothrombin cleavage by MASP-1 in a purified system which showed a dose- and time-response (Fig. 4b). In NCP, MASP-1 concentration correlated with generation of the fibrinogen cleavage product FPA, although it was again far less efficient than thrombin (Table 1, Fig. 4c). Using PT-DP, thrombin had similar effects on FPA generation compared with NCP, whereas MASP-1 showed only negligible FPA generation and failed to demonstrate a dose-response effect (Table 1, Fig. 4c). This suggests that in a plasma system, MASP-1-induced FPA cleavage is largely thrombin-dependent and secondary to prothrombin activation by MASP-1.

**Table 1:** Activation products of prothrombin, fibrinogen, and TAFI.

	NCP	PT-DP
Activation by	F1+2 (pmol/l)	F1+2 (pmol/l)
FXa 0.1 µg/ml (2.2 nM)	136417.0	Not applicable
MASP-1 20 µg/ml (440 nM)	689.3	Not applicable
MASP-1 10 µg/ml (220 nM)	461.2	Not applicable
MASP-1 5 µg/ml (110 nM)	299.9	Not applicable
MASP-1 2.5 µg/ml (55 nM)	232.2	Not applicable
No activation	136.0	Not applicable

Activation by	FPA (nmol/l)	FPA (nmol/l)
Thrombin 0.1 U/ml (2.8 nM)	556.6	516.9
MASP-1 20 µg/ml (440 nM)	18.7	4.2
MASP-1 10 µg/ml (220 nM)	12.3	4.0
MASP-1 5 µg/ml (110 nM)	7.8	4.0
MASP-1 2.5 µg/ml (55 nM)	4.8	4.0
No activation	<0.4	2.4

Activation by	TAFIa (nmol/l)	TAFIa (nmol/l)
Thrombin-thrombomodulin	360.3	549.1
MASP-1 50 µg/ml (1000 nM)	38.9	44.0
MASP-1 10 µg/ml (220 nM)	7.4	10.9
MASP-1 2 µg/ml (44 nM)	1.4	3.1

Activation products were measured in normal citrated plasma (NCP) and prothrombin-depleted plasma (PT-DP). Data are shown as mean (n = 2). TAFIa results are corrected for the non-activated samples, therefore no values for the non-activated samples are shown.

**Figure 5:**
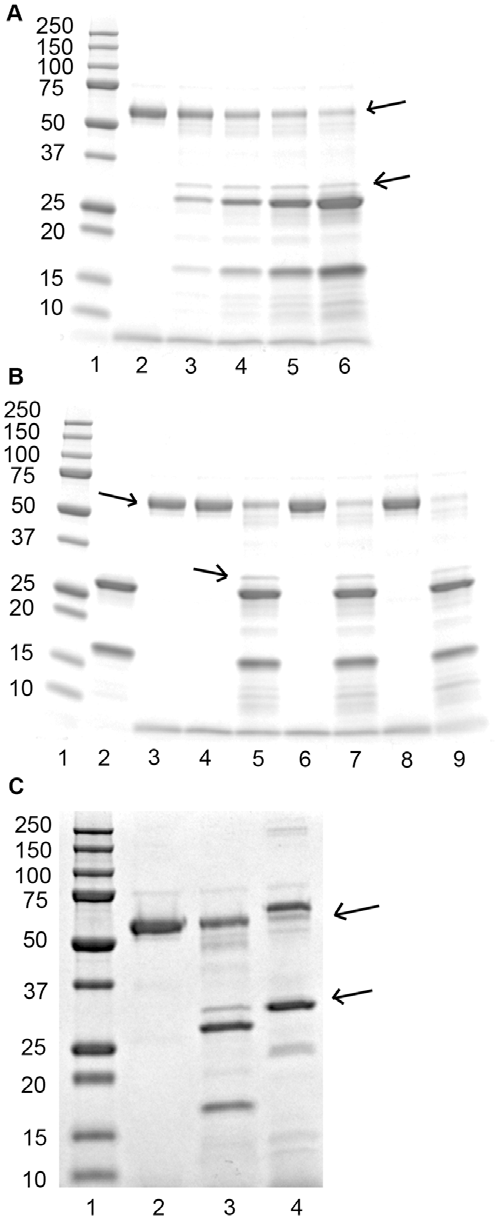
Cleavage of TAFI visualized by SDS-PAGE. (A) Dose-dependent effect of MASP-1 on cleavage of TAFI. Lane 1: Molecular weight marker. Lane 2: TAFI 200 µg/ml. Lane 3: TAFI+100 µg/ml MASP-1. Lane 4: TAFI+200 µg/ml MASP-1. Lane 5: TAFI+400 µg/ml MASP-1. Lane 6: TAFI+800 µg/ml MASP-1. (B) Time course of MASP-1 cleavage of TAFI. TAFI (200 µg/ml) was incubated at 37°C without or with MASP-1 (400 µg/ml). When TAFI was incubated without MASP-1, no cleavage occurred. Lane 1: Molecular weight marker. Lane 2: MASP-1 (fresh control). Lane 3: TAFI (fresh control). Lane 4: TAFI incubated for 30 min. Lane 5: TAFI+MASP-1 incubated for 30 min. Lane 6: TAFI incubated for 60 min. Lane 7: TAFI+MASP-1 incubated for 60 min. Lane 8: TAFI incubated for 120 min. Lane 9: TAFI+MASP-1 incubated for 120 min. (C) Physiological concentrations of TAFI (15 µg/ml) were incubated with either physiological concentrations of MASP-1 (15 µg/ml) or 1.7 U/ml (50 nM) thrombin and 3 µg/ml (58 nM) thrombomodulin. Lane 1: Molecular weight marker. Lane 2: TAFI. Lane 3: TAFI+MASP-1. Lane 4: TAFI+thrombin/thrombomodulin. In all three panels, arrows indicate full-length TAFI (58 kDa) and activated TAFIa (35 kDa).

In addition to prothrombotic factors, MASP-1 is capable of activating the antifibrinolytic protein TAFI. Using NCP, MASP-1 activated TAFI in a dose-dependent manner, although high concentrations were needed to show an effect and this was still around 10-fold lower than the activation achieved by the physiological TAFI activator thrombin-thrombomodulin complex (Table 1). MASP-1 also activated TAFI in PT-DP indicating that this process is not thrombin-dependent. Cleavage of TAFI by MASP-1 in a dose- (Fig. 5a) and time-dependent (Fig. 5b) manner was confirmed by SDS-PAGE. Again, MASP-1 was less efficient at cleaving TAFI than thrombin/thrombomodulin (Fig. 5c).

### Clot formation, structure and lysis using turbidimetric assays

MASP-1 induced clot formation in NCP, but not in PT-DP (not even at MASP-1 concentrations up to 100 µg/ml (2.2 µM)). This is in agreement with our FPA data above and suggests that in plasma environment fibrin formation is not directly induced by MASP-1 in the absence of prothrombin.

**Figure 6:**
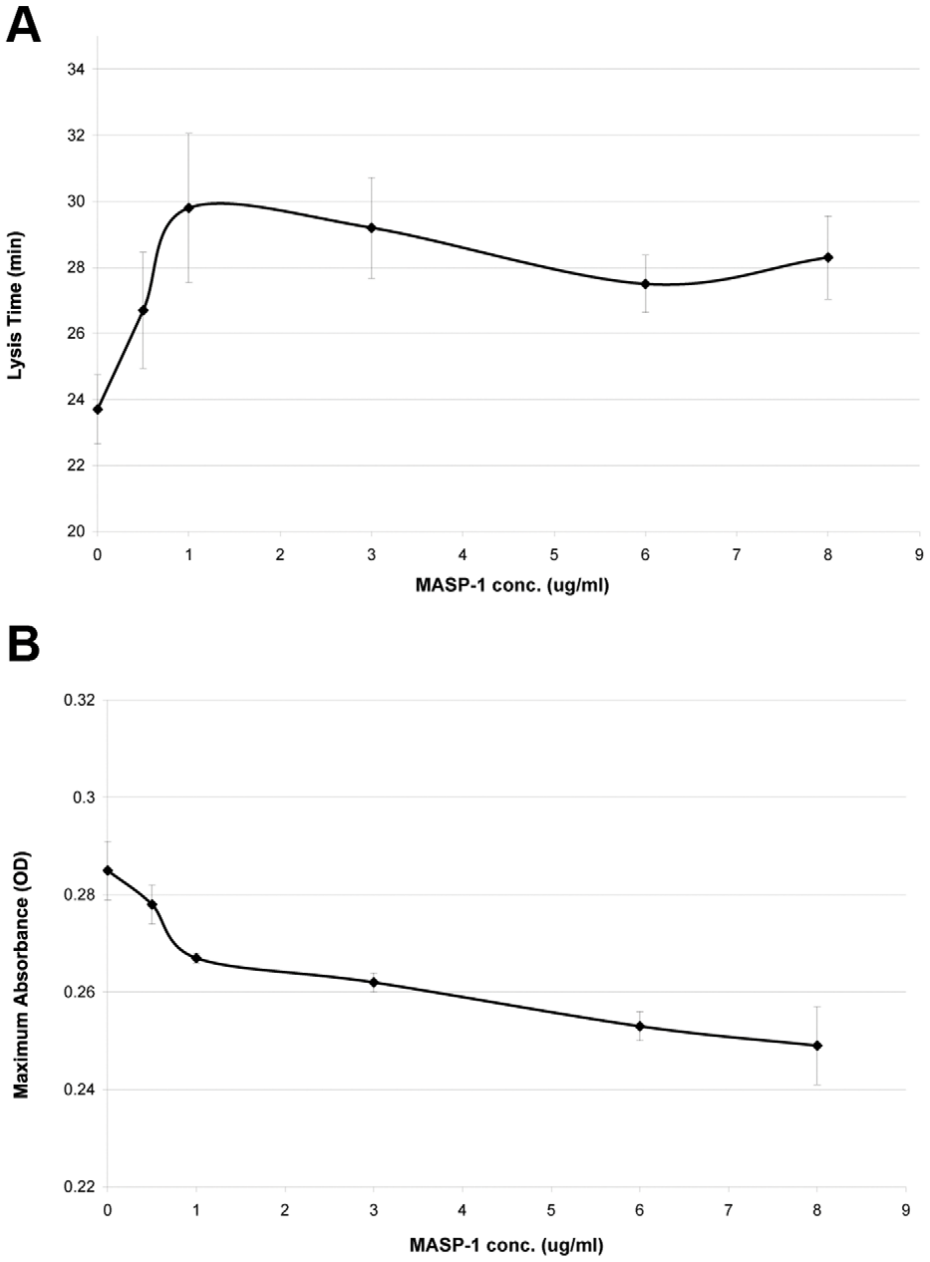
Effects of MASP-1 on plasma clot lysis time and maximum absorbance. Clot formation and lysis in normal plasma were investigated using a turbidimetric assay. Increasing MASP-1 concentrations were associated with longer clot lysis time (A) and lower maximum absorbance (B). Data points represent mean values from three experiments with error bars representing standard deviation.

MASP-1 had no clear influence on the initiation of clot formation in NCP assessed as lag time. However, addition of MASP-1 resulted in prolongation of lysis time, with maximum prolongation at 1 µg/ml (22 nM) but no additional effect at higher MASP-1 concentrations (Fig. 6a). Furthermore, MASP-1 had a dose-dependent effect on plasma clot maximum absorbance (Fig. 6b). MASP-1 concentrations between 0 and 8 µg/ml (176 nM) showed significant inverse correlation with maximum absorbance (Pearson correlation coefficient −0.964, p<0.001). These results suggested that higher MASP-1 concentrations were associated with either thinner fibers or reduction in clot density.

### Fibrin structure by SEM

**Figure 7:**
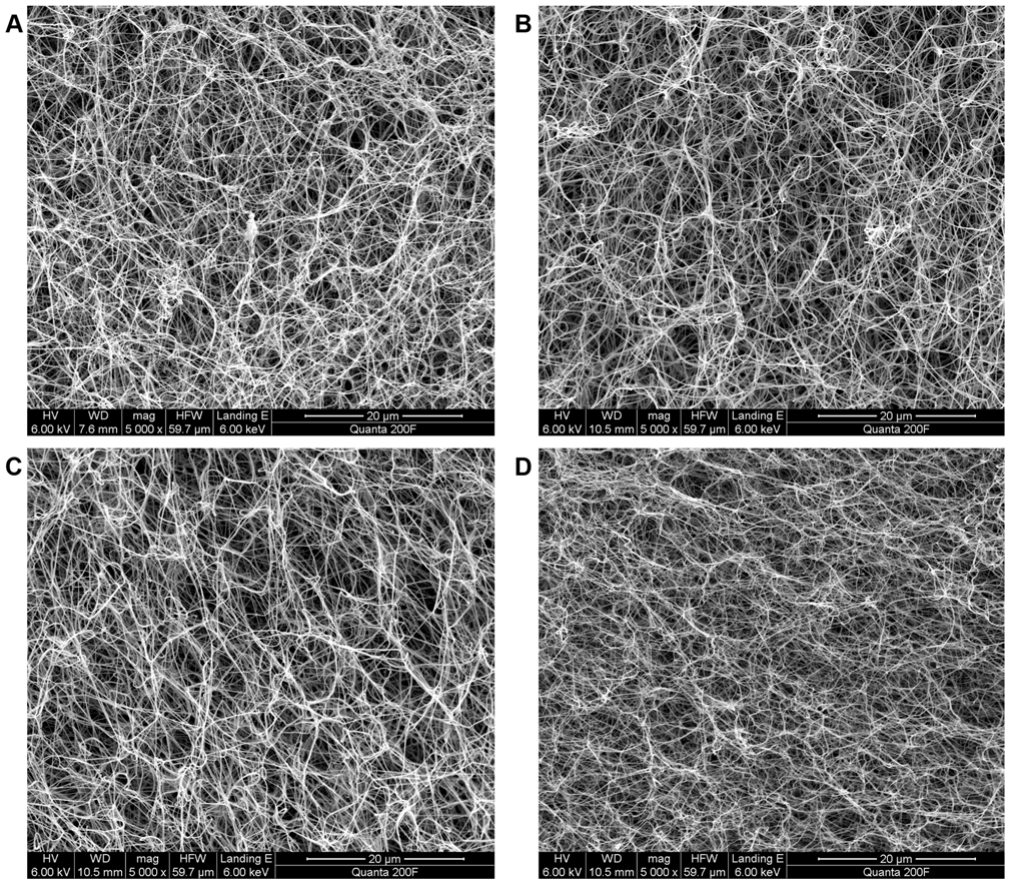
Fibrin structure assessed by SEM. Representative images of plasma clots prepared with (A) 1 µg/ml (22 nM) MASP-1, (B) 5 µg/ml (110 nM) MASP-1, (C) 10 µg/ml (220 nM) MASP-1, or (D) 0.88 U/ml (24 nM) of thrombin (magnification 5000×). Clots prepared in presence of MASP-1 showed a less dense structure with larger pores and thicker fibers.

Clots prepared with thrombin (Fig. 7d) were characterized by a dense fibrin network with thin fibers with a mean (SD) diameter of 102 (18) nm and small pores. In contrast, clots prepared in presence of MASP-1 (Fig. 7a–c) showed a less dense network with coarser fibers and larger pores. The mean (SD) fiber diameters of clots made with 1 µg/ml (22 nM), 5 µg/ml (110 nM) or 10 µg/ml (220 nM) of MASP-1 were 108 (17) nm, 120 (17) nm, and 134 (22) nm, respectively. Fiber diameters of MASP-1 clots were significantly higher than thrombin clots (p<.001), except for clots prepared with 1 µg/ml MASP-1 (p = .097).

### Role for MASP-1 in plasma clot formation

**Figure 8:**
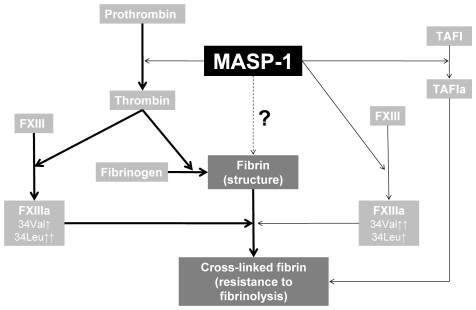
The proposed role of MASP-1 in plasma clot formation. MASP-1 induces fibrin clot formation by converting prothrombin to thrombin which then cleaves fibrinogen. MASP-1 activates FXIII and TAFI independently albeit at a lower efficiency than thrombin. Changes in clot structure and TAFI activation by MASP-1 may be responsible for prolonged lysis time of clots made in the presence of MASP-1. The finer arrows for MASP-1-dependent reactions, as compared with thrombin-dependent reactions, represent the fact that in plasma MASP-1 is less efficient compared with thrombin. The mechanisms underlying the impact of MASP-1 on fibrin structure remain to be elucidated.

Taken together, we propose the following role for MASP-1 in plasma clot formation, as summarized in Fig. 8. MASP-1 initiates fibrin clot formation by converting prothrombin to thrombin which then cleaves fibrinogen. MASP-1 activates FXIII independently from thrombin, having a greater effect on Val34 variant compared with Leu34, which is the opposite to thrombin. MASP-1 induces changes in fibrin clot structure that may be related to different plasma protein incorporation into the clot secondary to altered FXIII activation. This, together with direct TAFI activation by MASP-1, may be responsible for longer lysis time of clots formed following MASP-1 activation.

## Discussion

There is increasing interest in and mounting evidence for a role of the complement system in atherosclerosis [32]. In addition, more interactions between complement and coagulation are being discovered which could provide pathophysiological mechanisms linking inflammatory and thrombotic processes. The aim of the present work was to study interactions between MASP-1 and coagulation factors involved in fibrin formation in plasma and investigate whether MASP-1 is able to induce plasma clot formation independently from activation of coagulation pathways. Completely novel aspects of our work compared with earlier studies include the following: 1) experiments were performed in plasma environment, 2) we employed state of the art coagulation tests including sensitive activation tests and a turbidimetric clot formation and lysis test, 3) for the first time we studied the activation of both FXIII Val34 and Leu34 genetic variants by MASP-1, 4) we investigated for the first time MASP-1 effects on TAFI, and 5) we studied for the first time the structure of plasma clots made in presence of MASP-1 using SEM.

We showed that MASP-1 activates FXIII in plasma in a dose-dependent manner and independently from thrombin activity, albeit less efficiently compared to thrombin. MASP-1 specificity was further confirmed by inhibition of FXIII activation in the presence of C1-inhibitor. MASP-1 was slower at activating FXIII in plasma environment, with similar results observed in normal and prothrombin-depleted plasma, than in the purified system. This suggests that plasma contains factors that inhibit MASP-1-induced FXIII activation, but it also shows that MASP-1 activation of FXIII can occur directly and is not only thrombin-dependent. However, in plasma environment most FXIII activation by MASP-1 is probably mediated by thrombin and sufficient FXIIIa is generated to support normal fibrin cross-linking as shown by our cross-linking analysis. On the other hand, it is known that only traces (<1%) of FXIIIa are sufficient for fibrin cross-linking [33], so even in the absence of thrombin the FXIIIa directly generated by MASP-1 would be enough to cross-link any fibrin or other proteins. One striking observation was the difference in MASP-1-induced activation of FXIII genetic variants. The common FXIII Val34Leu polymorphism leads to altered FXIII activation by thrombin [28], [29]. The FXIII A-subunit residue at position P(4) from the thrombin cleavage site, corresponding to Val34, is important for recognition and binding of thrombin and hence FXIII activation [34], [35]. Due to its higher affinity to thrombin, the Leu34 variant is activated at a faster rate and paradoxically this variant protects from cardiovascular disease in most clinical studies [36]. Studies have struggled to explain this paradox and several hypotheses have been put forward including an interaction with fibrinogen levels and less efficient cross-linking of various proteins into the clot [29], [37], [38]. In our work, however, we have shown that MASP-1 activated the wild-type Val34 variant faster than the Leu34 variant, both in a purified system and in plasma environment. This suggests that i) MASP-1 activates FXIII in a different manner compared with thrombin possibly due to differences in substrate recognition and ii) interaction between the complement system and FXIII activation *in vivo* may contribute, at least in part, to the higher prevalence of thrombotic events with FXIII Val34 variant. Further work to elucidate the mechanisms behind this intriguing novel observation remains an area for future studies, including experiments at different fibrinogen concentrations since the protective effect of the FXIII Leu34 variant is strongest at high fibrinogen concentrations.

We also tested MASP-1-induced cleavage/activation of two other major thrombin substrates in plasma, fibrinogen and TAFI. Cleavage of the fibrinogen α-chain by MASP-1 has been suggested to occur at different sites compared with thrombin [19]. We have therefore tested whether MASP-1 induces the generation of FPA in plasma environment. In NCP, FPA generation correlated with MASP-1 concentration, but the quantities of FPA generated were much lower compared with thrombin. In PT-DP, negligible amounts of FPA were detected which failed to show a MASP-1 dose-response, suggesting that MASP-1-induced release of FPA relies on presence of prothrombin and possibly on thrombin generation. These findings support earlier data that fibrinogen cleavage by MASP-1 does not produce FPA [19] and suggest that direct cleavage of fibrinogen, and FXIII, by MASP-1 is probably of minor importance in plasma. The essential role of prothrombin is supported by our data demonstrating the inability of MASP-1 to induce fibrin clot formation in PT-DP in the turbidimetric assay, and the ability of MASP-1 to cleave prothrombin in a dose-dependent manner. Despite of the apparently better performance of MASP-1 than FXa in cleaving prothrombin in a purified system (Fig. 4b), it must be noted that FXa activity is underestimated in this system due to the lack of its prothrombinase complex cofactor FVa and phospholipids. In plasma (Table 1), prothrombin cleavage by MASP-1, similar to prothrombin cleavage by MASP-2 described by Krarup et al. [8], is much less effective compared to FXa, and would even be less effective when compared with the prothrombinase complex. However, thanks to its efficient amplification system, tiny amounts of generated thrombin can trigger significant activation of the coagulation cascade. We confirmed MASP-1 cleavage of prothrombin in a purified system, since this reaction had been excluded for MASP-1 earlier [8]. The discrepancy between our results and the study by Krarup et al. [8] may be explained by i) different experimental conditions, ii) difference in concentrations of tested proteins, and ii) different methods to detect prothrombin activation products. Krarup et al. used prothrombin at a final concentration of 110 µg/ml, MASP-1 at 1 µg/ml. We used a similar prothrombin concentration but MASP-1 was used at a wider range of physiological concentrations of 2.5–20 µg/ml. Also, Krarup et al. detected prothrombin cleavage by SDS-PAGE and Coomassie staining. Our method of detecting prothrombin fragment F1+2 by ELISA may be more sensitive.

In contrast to PT-DP, MASP-1 induced clot formation in NCP and an inverse correlation was detected between clot final turbidity and MASP-1 concentrations, indicating this protein modulates the final structure of the clot, which has not been documented before. It is accepted that lower maximum absorbance of clots made using purified systems indicates the formation of thinner fibers, but in plasma clots this is more complex as reduced maximum absorbance may be an indicator of thinner fibrin fibers and/or decreased clot density. MASP-1 has also affected clot structure as assessed by SEM. Clots made in the presence of MASP-1 had thicker fibers, were less dense and more porous compared with clots made after thrombin activation. Thus, results from the turbidimetric clotting assay and SEM both suggest that MASP-1 leads to a less dense clot structure with thicker fibrin fibers. It is well established that thrombin concentrations have a major impact on fibrin structure, with increasing thrombin concentrations resulting in a denser fibrin network with thinner fibers [39]–[41]. Intriguingly, MASP-1 seems to have the opposite effect as increasing MASP-1 concentrations were associated with less dense fibrin networks and thicker fibers, suggesting that a mechanism other than increased thrombin generation may be involved. So far, however, the way MASP-1 alters clot characteristics remains unclear. Although we show that fibrin formation in plasma is mainly thrombin-dependent, MASP-1 may interfere with prothrombin and fibrinogen resulting in slower production of fibrin monomers. Lower polymerization rates are known to produce a looser network with thicker fibers [39]. It has also been suggested that FPB release from the fibrinogen β-chain promotes lateral aggregation resulting in formation of thick fibers [42], [41]. Thus, additional FPB cleavage by MASP-1, as shown by Krarup et al. [19], may just have this effect. In addition, the observed differences in clot structure associated with MASP-1 may be related to different activation of FXIII and altered cross-linking of various proteins. These observations may also be in line with results by Gulla et al. [21] who suggested a similar clot composition but less cross-linking of clots made with MASP-1.

We show for the first time that MASP-1 is able to activate TAFI. This reaction is independent of thrombin generation as it was observed in both NCP and PT-DP and confirmed by SDS-PAGE. It should be noted, however, that MASP-1 activation of TAFI occurs at a much lower efficiency compared with thrombin/thrombomodulin. Nevertheless, additional TAFI activation by MASP-1 may explain the prolongation of lysis time associated with MASP-1 in the turbidimetric clotting and lysis assay, since TAFI is a major inhibitor of fibrinolysis. Another possible explanation for prolongation of lysis time may be the interaction between MASP-1 and FXIII which may lead to changes in fibrin cross-linking and cross-linking of antifibrinolytic proteins to fibrin, which in turn affects efficiency of fibrinolysis.

A less dense clot structure is generally associated with shorter lysis times, whereas we see longer lysis times of plasma clots made in the presence of MASP-1. Whether the profibrinolytic effect of a less dense network or the antifibrinolytic effect observed in the turbidimetric assay prevails *in vivo* remains to be investigated. If MASP-1 indeed led to prolongation of fibrinolysis, this would represent a further link between the complement system, in particular MBL pathway components, and changes in fibrin clot structure and prolongation of fibrinolysis, as our group has previously shown an independent association between complement C3 levels and prolongation of fibrinolysis [43]–[45].

A limitation of our study is the use of a recombinant mutant catalytic fragment of MASP-1 instead of the full-length molecule. We used this catalytic fragment for the following reasons: First of all, at present there is no appropriate method to purify native MASP-1 from plasma or serum and consequently several attempts to purify MASP-1 in sufficient amounts and free from contamination with other MASPs have failed. In addition, recombinant expression of full-length MASP-1 failed in all expression systems. In eukaryotic cells wild type MASP-1 is toxic. The recombinant MASP-1 constructs which could be expressed (e.g. in CHO cells) were mutants in the catalytic domain leading to reduced proteolytic efficiency. In contrast, the recombinant MASP-1 fragment we used in our study is the only preparation in the world which represents the wild type active human MASP-1. We are aware that, unlike the native protein, this catalytic fragment cannot form dimers or bind to MBL or ficolins and does not require an activation step and may therefore behave differently. However, the CUB-EGF-CUB domains, which are deleted in our fragment but present in the C1r, C1s and MASP proteins, are engaged in binding to the recognition molecules and consequently do not take part in substrate binding. The CCP modules, however, can contain exosites for substrate binding, as demonstrated for MASP-2 and C1s. Accordingly, our catalytic MASP-1 fragment contains both CCP modules. In addition, recombinant catalytic fragments of C1r, a MASP homolog, have been shown to retain the catalytic efficiency and substrate specificity of the entire molecule [9]. Endogenous MASP-1 circulates in plasma as a zymogen. The active recombinant fragment, rMASP-1 CCP1-CCP2-SP, we used in our study might activate the endogenous zymogen. In addition, it might also activate MASP-2 as recently shown [13]. However, in both cases the measured effects would still be due to MASP-1. Taken together, we do believe that the catalytic MASP-1 fragment we have used in our study represents the best model protein currently available to achieve our main goal which was to investigate MASP-1 catalytic properties towards coagulation proteins in plasma.

In summary, we used an array of coagulation factor activation and plasma clotting assays as well as SEM of plasma clots to show that MASP-1, albeit not able to induce fibrin clot formation completely independently of coagulation activation, interacts with plasma clot formation on different levels and influences the resulting fibrin structure. MASP-1 activates FXIII independently from thrombin and has a differential effect on FXIII Val34 and Leu34 variants. On the other hand, fibrinogen-to-fibrin conversion by MASP-1 is related to thrombin generation through prothrombin activation. Fibrin clot formation can be initiated by MASP-1 in plasma and this has an effect on lysis time, which may be related to changes in clot structure, activation of FXIII and TAFI, or other unknown factors. Our results support the first *in vivo* findings for a role of MASP-1 in coagulation and thrombus formation [22], [23], but further investigations to elucidate the precise mechanisms and physiological relevance in humans are needed. Nevertheless, our results are consistent with a possible role for MASP-1 in plasma fibrin clot formation and fibrinolysis, which may have future clinical implications.

## References

[1] Opal SM (2000). Phylogenetic and functional relationships between coagulation and the innate immune response.. Crit Care Med.

[2] Markiewski MM, Nilsson B, Nilsson Ekdahl K, Mollnes TE, Lambris JD (2007). Complement and coagulation: strangers or partners in crime?. Trends Immunol.

[3] Amara U, Flierl MA, Rittirsch D, Klos A, Chen Hui (2010). Molecular intercommunication between the complement and coagulation systems.. J Immunol.

[4] Hajela K, Kojima M, Ambrus G, Wong KHN, Moffatt BE (2002). The biological functions of MBL-associated serine proteases (MASPs).. Immunobiology.

[5] Gál P, Barna L, Kocsis A, Závodszky P (2007). Serine proteases of the classical and lectin pathways: similarities and differences.. Immunobiology.

[6] Terai I, Kobayashi K, Matsushita M, Fujita T (1997). Human serum mannose-binding lectin (MBL)-associated serine protease-1 (MASP-1): determination of levels in body fluids and identification of two forms in serum.. Clin Exp Immunol.

[7] Thiel S, Jensenius JC, Jensen L, Degn S (2011). Normal and acute phase variations of MASP-1, an enzyme associated with humoral pattern recognition molecules.. Mol Immunol.

[8] Krarup A, Wallis R, Presanis JS, Gál P, Sim RB (2007). Simultaneous activation of complement and coagulation by MBL-associated serine protease 2.. PLoS One.

[9] Ambrus G, Gál P, Kojima M, Szilágyi K, Balczer J (2003). Natural substrates and inhibitors of mannan-binding lectin-associated serine protease-1 and -2: a study on recombinant catalytic fragments.. J Immunol.

[10] Schwaeble WJ, Lynch NJ, Clark JE, Marber M, Samani NJ (2011). Targeting of mannan-binding lectin-associated serine protease-2 confers protection from myocardial and gastrointestinal ischemia/reperfusion injury.. Proc Natl Acad Sci USA.

[11] Møller-Kristensen M, Thiel S, Sjöholm A, Matsushita M, Jensenius JC (2007). Cooperation between MASP-1 and MASP-2 in the generation of C3 convertase through the MBL pathway.. Int Immunol.

[12] Takahashi M, Iwaki D, Kanno K, Ishida Y, Xiong J (2008). Mannose-binding lectin (MBL)-associated serine protease (MASP)-1 contributes to activation of the lectin complement pathway.. J Immunol.

[13] Kocsis A, Kékesi KA, Szász R, Végh BM, Balczer J (2010). Selective inhibition of the lectin pathway of complement with phage display selected peptides against mannose-binding lectin-associated serine protease (MASP)-1 and -2: Significant contribution of MASP-1 to lectin pathway activation.. J Immunol.

[14] Takahashi M, Ishida Y, Iwaki D, Kanno K, Suzuki T (2010). Essential role of mannose-binding lectin-associated serine protease-1 in activation of the complement factor D.. J Exp Med.

[15] Banda NK, Takahashi M, Levitt B, Glogowska M, Nicholas J (2010). Essential role of complement mannose-binding lectin-associated serine proteases-1/3 in the murine collagen antibody-induced model of inflammatory arthritis.. J Immunol.

[16] Dobó J, Major B, Kékesi KA, Szabó I, Megyeri M (2011). Cleavage of kininogen and subsequent bradykinin release by the complement component: Mannose-binding lectin-associated serine protease (MASP)-1.. PLoS One.

[17] Presanis JS, Hajela K, Ambrus G, Gál P, Sim RB (2004). Differential substrate and inhibitor profiles for human MASP-1 and MASP-2.. Mol Immunol.

[18] Dobó J, Harmat V, Beinrohr L, Sebestyén E, Závodszky P (2009). MASP-1, a promiscuous complement protease: structure of its catalytic region reveals the basis of its broad specificity.. J Immunol.

[19] Krarup A, Gulla KC, Gál P, Hajela K, Sim RB (2008). The action of MBL-associated serine protease 1 (MASP1) on factor XIII and fibrinogen.. Biochim Biophys Acta.

[20] Megyeri M, Makó V, Beinrohr L, Doleschall Z, Prohászka Z (2009). Complement protease MASP-1 activates human endothelial cells: PAR4 activation is a link between complement and endothelial function.. J Immunol.

[21] Gulla KC, Gupta K, Krarup A, Gál P, Schwaeble WJ (2010). Activation of mannan-binding lectin-associated serine proteases leads to generation of a fibrin clot.. Immunology.

[22] Takahashi K, Chang WC, Takahashi M, Pavlov V, Ishida Y (2011). Mannose-binding lectin and its associated proteases (MASPs) mediate coagulation and its deficiency is a risk factor in developing complications from infection, including disseminated intravascular coagulation.. Immunobiology.

[23] La Bonte LR, Pavlov VI, Tan YS, Takahashi K, Takahashi M (2012). Mannose-binding lectin-associated serine protease-1 is a significant contributor to coagulation in a murine model of occlusive thrombosis.. J Immunol.

[24] Fatah K, Silveira A, Tornvall P, Karpe F, Blombäck M (1996). Proneness to formation of tight and rigid fibrin gel structures in men with myocardial infarction at a young age.. Thromb Haemost.

[25] Collet JP, Allali Y, Lesty C, Tanguy ML, Silvain J (2006). Altered fibrin architecture is associated with hypofibrinolysis and premature coronary atherothrombosis.. Arterioscler Thromb Vasc Biol.

[26] Collet JP, Park D, Lesty C, Soria J, Soria C (2000). Influence of fibrin network conformation and fibrin fiber diameter on fibrinolysis speed: dynamic and structural approaches by confocal microscopy.. Arterioscler Thromb Vasc Biol.

[27] Ajjan R, Lim BCB, Standeven KF, Harrand R, Dolling S (2008). Common variation in the C-terminal region of the fibrinogen β-chain: effects on fibrin structure, fibrinolysis and clot rigidity.. Blood.

[28] Kohler HP, Ariëns RAS, Whitaker P, Grant PJ (1998). A common coding polymorphism in the factor XIII A-subunit gene (FXIIIVal34Leu) affects cross-linking activity.. Thromb Haemost.

[29] Ariëns RA, Lai TS, Weisel JW, Greenberg CS, Grant PJ (2002). Role of factor XIII in fibrin clot formation and effects of genetic polymorphisms.. Blood.

[30] Bajzar L, Morser J, Nesheim M (1996). TAFI, or plasma procarboxypeptidase B, couples the coagulation and fibrinolytic cascades through the thrombin-thrombomodulin complex.. J Biol Chem.

[31] Carter AM, Cymbalista CM, Spector TD, Grant PJ (2007). Heritability of clot formation, morphology, and lysis: the EuroCLOT study.. Arterioscler Thromb Vasc Biol.

[32] Speidl WS, Kastl SP, Huber K, Wojta J (2011). Complement in atherosclerosis: friend or foe?. J Thromb Haemost.

[33] Mikkola H, Muszbek L, Laiho E, Syrjälä M, Hämäläinen E (1997). Molecular mechanism of a mild phenotype in coagulation factor XIII (FXIII) deficiency: a splicing mutation permitting partial correct splicing of FXIII A-subunit mRNA.. Blood.

[34] Trumbo TA, Maurer MC (2002). Thrombin hydrolysis of V29F and V34L mutants of factor XIII (28–41) reveals roles of the P(9) and P(4) positions in factor XIII activation.. Biochemistry.

[35] Jadhav MA, Lucas RC, Goldsberry WN, Maurer MC (2011). Design of factor XIII V34X activation peptides to control ability to interact with thrombin mutants.. Biochim Biophys Acta.

[36] Muszbek L, Bereczky Z, Bagoly Z, Shemirani AH, Katona E (2010). Factor XIII and atherothrombotic diseases.. Semin Thromb Hemost.

[37] Lim BC, Ariens RA, Carter AM, Weisel JW, Grant PJ (2003). Genetic regulation of fibrin structure and function: complex gene-environment interactions may modulate vascular risk.. Lancet.

[38] Bereczky Z, Balogh E, Katona E, Pocsai Z, Czuriga I (2007). Modulation of the risk of coronary sclerosis/myocardial infarction by the interaction between factor XIII subunit A Val34Leu polymorphism and fibrinogen concentration in the high risk Hungarian population.. Thromb Res.

[39] Ryan EA, Mockros LF, Weisel JW, Lorand L (1999). Structural origins of fibrin clot rheology.. Biophys J.

[40] Weisel JW (2004). The mechanical properties of fibrin for basic scientists and clinicians.. Biophys Chem.

[41] Wolberg AS (2007). Thrombin generation and fibrin clot structure.. Blood Reviews.

[42] Blombäck B, Hessel B, Hogg D, Therkildsen L (1978). A two-step fibrinogen-fibrin transition in blood coagulation.. Nature.

[43] Schroeder V, Carter AM, Dunne J, Mansfield MW, Grant PJ (2010). Proinflammatory and hypofibrinolytic phenotype in healthy first-degree relatives of patients with type 2 diabetes.. J Thromb Haemost.

[44] Howes JM, Richardson VR, Smith KA, Schroeder V, Somani R (2012). Complement C3 is a novel plasma clot component with anti-fibrinolytic properties.. Diab Vasc Dis Res.

[45] Hess K, Alzahrani S, Mathai M, Schroeder V, Carter AM (2012). A novel mechanism for hypofibrinolysis in diabetes: The role of Complement C3.. Diabetologia.

